# Multistakeholder Recommendations for Supporting Patients and Families Transitioning From Paediatric to Adult Congenital Heart Disease Care

**DOI:** 10.1016/j.cjcpc.2023.08.001

**Published:** 2023-08-18

**Authors:** Emily K. Hyde, Annette S.H. Schultz, Robin Ducas, Reeni Soni, Holly Bekkering, Dawn Barker, Andrea Klippenstein, Mudra G. Dave, Chloe Frechette, Joanne St. Goddard-Frechette, Lori Lester, Shelly Mclarty, Anna M. Chudyk

**Affiliations:** a Winnipeg Regional Health Authority, Winnipeg, Manitoba, Canada; b College of Nursing, Rady Faculty of Health Sciences, University of Manitoba, Winnipeg, Manitoba, Canada; c Institute of Cardiovascular Sciences, St. Boniface General Hospital Albrechtsen Research Centre, Winnipeg, Manitoba, Canada; d Section of Cardiology, Department of Internal Medicine, University of Manitoba, Winnipeg, Manitoba, Canada; e Children’s Heart Centre, Department of Pediatrics, University of Manitoba, Winnipeg, Manitoba, Canada; f Health Sciences Center, Winnipeg, Manitoba, Canada; g St Boniface Hospital, Winnipeg, Manitoba, Canada; h Faculty of Kinesiology and Recreation Management, University of Manitoba, Winnipeg, Manitoba, Canada; i Patient partner

## Abstract

**Background:**

Transitioning from paediatric to adult congenital heart disease (CHD) care is a high-risk time for being lost to follow-up. Existing CHD transition programmes have not included patients, caregivers, and health care providers as partners in their development. This study aimed to develop recommendations for a CHD transition programme driven by lived and clinical experiences.

**Methods:**

We used a multilevel participatory process that engaged adult and paediatric people living with CHD, their caregivers, and CHD health care providers as members of the research team. We also consulted members of these stakeholder groups through a series of 3 virtual workshops that culminated in the generation of recommendations for the essential components of a CHD transition programme.

**Results:**

The Transition Essentials recommendations inform what information, education, or support is required, who should provide it, and when and how it should be provided. Information, education, and support for self-management and knowledge are required for people living with CHD. Caregivers require information, education, and support to build capacity in people living with CHD and navigate their new role in their loved ones’ life. The health care team should provide this information, education, and support with peer support options when people living with CHD are 15-22 years of age. This information, education, and support should be individualized, navigate limitations, build over time, have multimodal options, and be available virtually or in person.

**Conclusions:**

Engaging those with lived and clinical expertise to develop recommendations for the essential components of a CHD transition programme provides important insights missing from previous studies.

## Résumé

**Contexte:**

La transition des personnes qui vivent avec une cardiopathie congénitale (CC) entre les soins pédiatriques et les soins destinés aux adultes constitue une période où le risque de perte de vue est élevé. Les programmes de transition existants n’ont pas été élaborés avec la participation des patients, des aidants ou des fournisseurs de soins de santé. La présente étude visait à mettre en place des recommandations fondées sur la réalité des personnes concernées et sur l’expérience clinique pour les programmes de transition en contexte de CC.

**Méthodologie:**

Nous avons fait appel à un processus participatif à plusieurs niveaux dans lequel des enfants et des adultes vivant avec la CC, des aidants et des fournisseurs de soins de santé du domaine de la CC ont été impliqués comme membres de l’équipe de recherche. Nous avons également mené des consultations auprès de ces groupes d’intervenants dans une série de trois ateliers virtuels qui ont mené à la rédaction de recommandations sur les composantes essentielles d’un programme de transition pour les personnes vivant avec une CC.

**Résultats:**

Les recommandations portant sur les impératifs d’une transition réussie énoncent les renseignements, la formation et le soutien nécessaires ainsi que les intervenants qui devraient les offrir, de quelle façon et à quel moment. Les personnes qui vivent avec une CC ont besoin de renseignements, de formation et de soutien pour l’autoprise en charge et l’accès aux connaissances. Quant aux aidants, ils ont aussi besoin de renseignements, de formation et de soutien pour mieux outiller les personnes qui vivent avec une CC et pour mieux comprendre leur nouveau rôle dans la vie de leur proche. Il conviendrait que les professionnels de la santé soient ceux qui offrent ces ressources, lesquelles devraient être personnalisées, tenir compte des lacunes à combler, être cumulatives, offrir des options multimodales et être accessibles en personne ou virtuellement. Les personnes de 15 à 22 ans qui vivent avec une CC devraient également avoir la possibilité de s’entraider.

**Conclusions:**

La participation des personnes qui ont une expertise ancrée dans la réalité et une expertise clinique afin de formuler des recommandations sur les éléments essentiels d’un programme de transition pour les personnes qui vivent avec une CC a permis d’obtenir des renseignements intéressants qui ne se trouvaient pas dans les études antérieures.

Congenital heart disease (CHD) is a broad spectrum of structural abnormalities of the heart that are present at birth.^1^ With improvements in interventions and care of children with CHD, there are a growing number of adults with CHD.^1^^,^^2^ The emergence of this population has resulted in specialized adult CHD medical care, but our knowledge of how to support young adults with transitioning their health care from the paediatric CHD care context to adult CHD care remains relatively unexplored.^2^ *Transfer* of care is the single occurrence of care changing from the paediatric to the adult setting and occurs variably in age and process.^3^ *Transition* of care is an ongoing process that bridges adolescence and young adulthood and prepares patients to understand and self-manage their health condition.^3^

Although leaving one care team for another might seem simple, transition is a time of high risk of being lost to follow-up or having a lapse in care^4^^,^^5^ as it involves navigating a new health care team^2^^,^^5^ and possibly health care facility.^5^ In many cases, patients are also now expected to manage this independently, as the burden of responsibility shifts from unpaid caregivers (eg, parents and guardians) to patients.^2^ Caregivers also undergo a transition in parallel with patients with similar and unique needs, such as learning to support their loved one in establishing their independence and understanding where they fit within the new care team.^2^

An emerging body of literature has begun to explore programmes to support transitions in CHD care.^6^, ^7^, ^8^, ^9^ There is emerging evidence of the value of transition programmes, including improved knowledge and self-management skills.^8^^,^^9^ However, despite the aim of focusing on the experience of care,^2^ to date, research has mainly involved patients with CHD, their caregivers, and health care providers as research subjects. Although opinions and preferences can be included in the design of a transition programme through survey and qualitative research, they are integrated through the researchers rather than directly through partnering with those with lived and clinical expertise. This excludes the opportunity to directly leverage lived and clinical expertise as sources of knowledge and integrate these into the design of transitions in CHD care programmes. Therefore, this study aimed to leverage patient, caregiver, and health care provider experiences to develop recommendations for a transition programme driven by lived and clinical experiences.

## Methods

### Guiding framework

Our participatory codesign approach to engaging patients, caregivers, and health care providers in health service redesign was informed by the Canadian Institutes of Health Research Strategy for Patient-Oriented Research Patient Engagement Framework,^10^ our scoping review of models and frameworks of patient engagement in health services research,^11^^,^^12^ and a scoping review of patient engagement for improving quality of care.^13^ Specifically, the Canadian Institutes of Health Research Strategy for Patient-Oriented Research Patient Engagement Framework provided guiding concepts, key principles, and outcomes to consider in engagement;^10^ however, it did not describe actual actions for engagement. The aforementioned scoping reviews^11^, ^12^, ^13^ expanded on key principles and outcomes of engagement and informed the activities we undertook to engage patients, caregivers, and clinicians.

The operationalization of our guiding framework of engagement was informed by the International Association for Public Participation Spectrum of Public Participation.^14^ This spectrum describes engagement along a continuum of increasing impact on research decision-making, from “inform” to “empower.”^14^ For example, collaborate is in the middle of the spectrum and is described as “to partner with the public in each aspect of the decision, including the development of alternatives and the identification of the preferred solution.”^14^ We engaged 2 individuals with lived experience of CHD of a successful transition to adult CHD (CF and JSGF, herein referred to as patient partners) and 4 health care providers with clinical expertise in CHD care (RD, RS, HB, and DB, herein referred to as clinical partners) at the level of collaborate^14^ throughout the study. The patient and clinical partners contributed expertise as members of our research team through meetings, providing feedback on the grant application and workshop plans, and to this article. They also participated in the workshops described below, sharing their lived and clinical perspectives with the other participants. The patient partners wished to contribute their experiences in this way. The clinical partners are part of small teams, so the limited number of health care providers required their participation in the workshops. In addition, we engaged 12 patients and caregivers, and 1 health care provider at the level of consult, which is described as gaining public feedback.^14^ Engaging with stakeholders (ie, patients, caregivers, and health care providers) at multiple levels enabled our study design and conduct to be directly guided by patient, caregiver, and health care provider experiences and expertise. Consulting additional stakeholders allowed a broader range of perspectives to inform the recommendations generated through the workshops described below. Table 1 summarizes these stakeholders’ overarching roles and our goals for engagement by the level of engagement.

**Table 1:** Summary of stakeholders’ overarching roles and our goals for engagement by level of engagement

Level of Engagement	Overarching Roles	Goals for Engagement
Involve	Provide insights and recommendations that the research team integrated into the study to ensure the lived experience was consistently considered and understood	To ensure the project included patient, caregiver, and healthcare provider-identified priorities and outcomes and was co-driven by their voices.
Consult	Share lived and clinical expertise through facilitated workshops	To leverage their lived and clinical expertise to generate patient, caregiver, and healthcare provider-orientated insights for successful transition and transfer from pediatric to adult specialized CHD care.

Note: the overarching roles and goals for engagement are informed by the IAP2 Spectrum of Public Participation (2018).^14^

### Recruitment and compensation

This study was conceptualized by 3 members of the research team (EKH, ASHS, and AMC). Between November 2021 and February 2022, they then recruited the study’s 2 patient partners and 4 clinical partners to engage in the development of a grant application to support this work. Patient partners were identified by reviewing patients who had successfully transitioned to adult CHD care and continued to be cared for by local adult CHD specialists. Clinical partners were identified through discussions with the local paediatric and adult CHD specialist health care providers. See Box 1 for an overview of the local context of CHD care. On receipt of funding and following university and site ethical approvals, people living with CHD, their caregivers, and the final health care provider were recruited from the paediatric and adult CHD clinics for the consultation roles. As described in a separate study,^15^ recruitment was focused on those who represented the shared experience of receiving CHD specialized care or being the caregiver or health care provider of someone receiving CHD specialized care in Manitoba. Specifically, we aimed to recruit stakeholders from each of the following groups: (1) paediatric patients (aged 15-18 years) who had not yet transitioned to adult care, (2) caregivers of paediatric patients who had not yet transitioned to adult care, (3) young adult patients (aged 19-25 years) who had successfully transitioned to adult care, (4) caregivers of young adult patients who had successfully transitioned to adult care, and (5) health care providers from paediatric and adult specialized CHD care. The desired size of each group was 5 to facilitate discussion and participation.^16^ Recruitment took place from September to December 2022.

**Box 1 Local context**

• The paediatric congenital heart disease (CHD) clinic is located at a separate hospital from the adult CHD clinic.
• Since 2018, one adult CHD physician has attended the paediatric CHD clinic 4 times yearly for a transition-focused clinic.
• In 2021, the adult CHD clinic was provided with a nurse who also attends the transition-focused clinic.
• Patients with moderate-to-complex CHD and their caregivers are invited to one of these clinics before transferring from paediatric to adult CHD care.
• The physicians assess the patient together and answer any medical questions.
• Nurses provide information, including their most recent diagnostic test results, clinic notes, a map of the new hospital, and self-care.
• Families are given the option to book an in-person appointment with the paediatric nurse clinician to review any transition-related concerns. They are also encouraged to reach out to either the paediatric or adult teams for help navigating the adult system.
• After the transition-focused clinic, patients are seen by the adult CHD team for their next appointment.
• There are no additional transition-specific appointments or education consistently provided.

Patient partners were each compensated CAD$500 for their participation in creating the grant application and another $500 for their participation in meetings and workshop preparation. Clinical partners were not compensated as the grant applications, meetings, and workshop preparation occurred during regular work hours. Patients and caregivers were compensated $100 for participating in the consultation workshops.^17^ Health care providers were provided with a $25 honorarium for their participation. All participants, including patient partners, clinical partners, and those participating at the level of consult, were offered the opportunity to coauthor this article. Reporting of this work was informed by the Guidance for Reporting Involvement of Patients and the Public 2 (GRIPP2) Long Form.^18^ Ethics approval was obtained from the University of Manitoba Research Ethics Board, the Shared Health Research and Innovation Office, and the St. Boniface Hospital Research Review Committee.

### Consultation workshops

**Figure 1:**
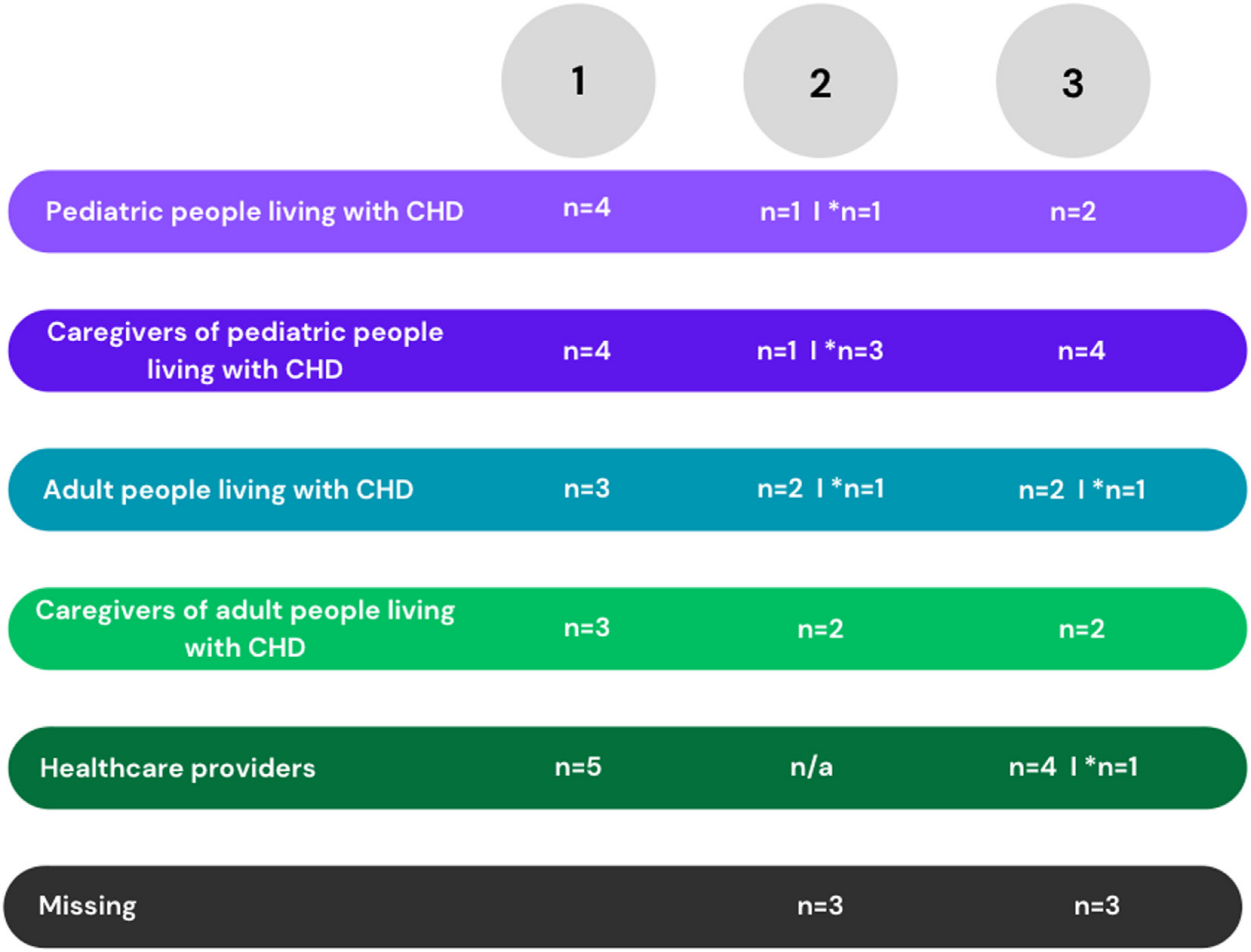
The number of people who engaged in each workshop by the stakeholder group. ∗Indicates individual feedback due to being unable to attend workshop. CHD, congenital heart disease.

Three consultation workshops were held virtually via Microsoft Teams between October 2022 and January 2023 (Figure 1). All were recorded and transcribed verbatim and also documented through a notetaker. Based on our previous work,^19^^,^^20^ each consultation workshop had a distinct but interconnected aim. Specifically, the first consultation workshop aimed to discuss concerns, knowledge, gaps, and opportunities related to transitions in care to begin to develop recommendations for transitions in CHD care. It met this aim through separate facilitated sessions held with each stakeholder group (n_total_ sessions = 5) that were guided by discussion questions specific to the stage and role of each stakeholder group (Table 2). The second consultation workshop aimed to review and build on the preliminary recommendations identified in the first consultation workshop to further develop recommendations for closing the gaps and optimizing the opportunities related to transitions in care. It met this aim through a facilitated session that included all patients and caregivers from paediatric and adult groups (Figure 1), guided by the preliminary recommendations and potential tools to support the transition in CHD care. The second and third workshop discussions were not driven by questions but by conversations reviewing the recommendations, so exact questions cannot be shared. The tools were selected based on suggestions from participants in the first consultation workshop and aimed to provide tangible examples of ideas shared by the participants. For instance, caregivers suggested a checklist of topics to cover related to self-management, so the TRANSITION-Q^21^ and the Transition Readiness Assessment Questionnaire^22^ were shared by EKH as examples of available tools that can be used to identify needs related to transition. Virtual options for delivery were also suggested, so the I Heart Change website^23^ and the MyTransition phone application (app) were shared by EKH as an example. The third consultation workshop involved all stakeholders from the first consultation workshop to arrive at essential recommendations for transitions from paediatric to adult CHD care. It met this aim through a facilitated session with all stakeholder groups (n_total_ groups = 5, Figure 1) that further developed the recommendations and tools to support a lived and clinical-experience–informed CHD transition programme.

**Table 2:** Overview of aims, activities, and outputs by workshop

Workshop number and duration	Attendees	Aim	Discussion questions/topics
1, 2 h	Separate workshops (5) for each stakeholder group	To discuss questions 1, 2, and 3 with each stakeholder group separately	1. “What are you worried about related to the upcoming transition to adult care”; OR “What helped during the transition to adult care”; OR “What is your experience of concerns related to transitions in care”? 2. “What do you want to know related to the upcoming transition to adult care”; OR “What do you wish you had known before the transition to adult care”; OR “What do you get asked about around the time of transition in care”? 3. What and where are the gaps in transitions from paediatric to adult CHD care and where you would like it to be in 5 years? Where do you want it to be/wish it would have been for you?
2, 2 h	All people living with CHD and caregivers	To review and discuss the preliminary recommendations collated from all stakeholder groups’ findings from the previous workshops	Group discussion of the preliminary recommendations and their alignment with lived experiences: *Preliminary recommendations:* The What • Build capacity in the patients to take charge of own care/life • Coaching of parents/caregivers in how to help build capacity The Who • Health care team • Peer support The When • Start at 15/16 • Continue to 22/23 The How • Tailor transition to needs—options to select • HCPs provide education/information • Checklist of topics to cover before transition • Shift conversations from “can’t” focus • Virtual resources
3, 2 h	All people living with CHD, caregiver, and health care providers	To bring together all stakeholders to arrive at recommendations essential for transitions from paediatric to adult congenital care	Group discussion of the revised recommendations and their alignment with lived experiences: *Revised recommendations:* The What • Build capacity in the patients to take charge of own care/life • Coaching of parents/caregivers in how to help build capacity The Who • Health care team • Peer support The When • Start at 15/16 • Continue to 22/23 The How • Tailor transition to needs via the use of a questionnaire • Shift conversations from “can’t” focus • Virtual resources—website and app • In person and virtual appointments

CHD, congenital heart disease; HCP, health care professional.

### Analysis

As described in a separate study,^15^ the purpose of engagement is to listen to, learn from, and apply stakeholder experiences, not analyse them as qualitative data sources. For this reason, typical qualitative methods are not used to analyse transcripts of discussions; instead, the information is collated and presented as recommendations.^15^ After completion of the 5 separate sessions for the first consultation workshop, findings from the workshop transcripts were collated by the first author (EKH) into recommendations related to what information, education, or support is important to share, by whom information or education should be shared, when information, education, or support should be shared, and how information, education, or support should be shared.^13^ These recommendations and the tools described above were then shared with the patient partners for their feedback on the alignment with their experiences. The recommendations and examples of tools were then discussed with all patients and caregivers at the second consultation workshop. After the completion of the second consultation workshop, EKH further revised the recommendations based on the workshop transcripts and shared them with all workshop participants for discussion at the third consultation workshop. There were several changes to the recommendations after the second workshop to ensure that the recommendations reflected workshop participants’ experiences. For example, the first author inferred from the transcripts that peer support would not be beneficial; however, the patients and caregivers clarified the context that peer support would be helpful so this was added. At the third consultation workshop, all patients, caregivers, and health care providers discussed the recommendations and examples of tools, focusing on identifying how the recommendations could be integrated into clinical practice and by which specific health care providers.

## Results

A total of 25 individuals (5 per group) agreed to participate in the consultation workshops. However, as displayed in Figure 1, attendance varied among workshops. Notably, after agreeing to participate in the study, 6 individuals did not respond to any subsequent meeting requests or return emails or telephone calls throughout the study. They did not attend any actual workshop sessions. Further, several stakeholders could not attend workshop sessions 2 and 3, so they provided feedback to EKH individually through one-on-one conversations that followed the same format as the workshop sessions.

**Figure 2:**
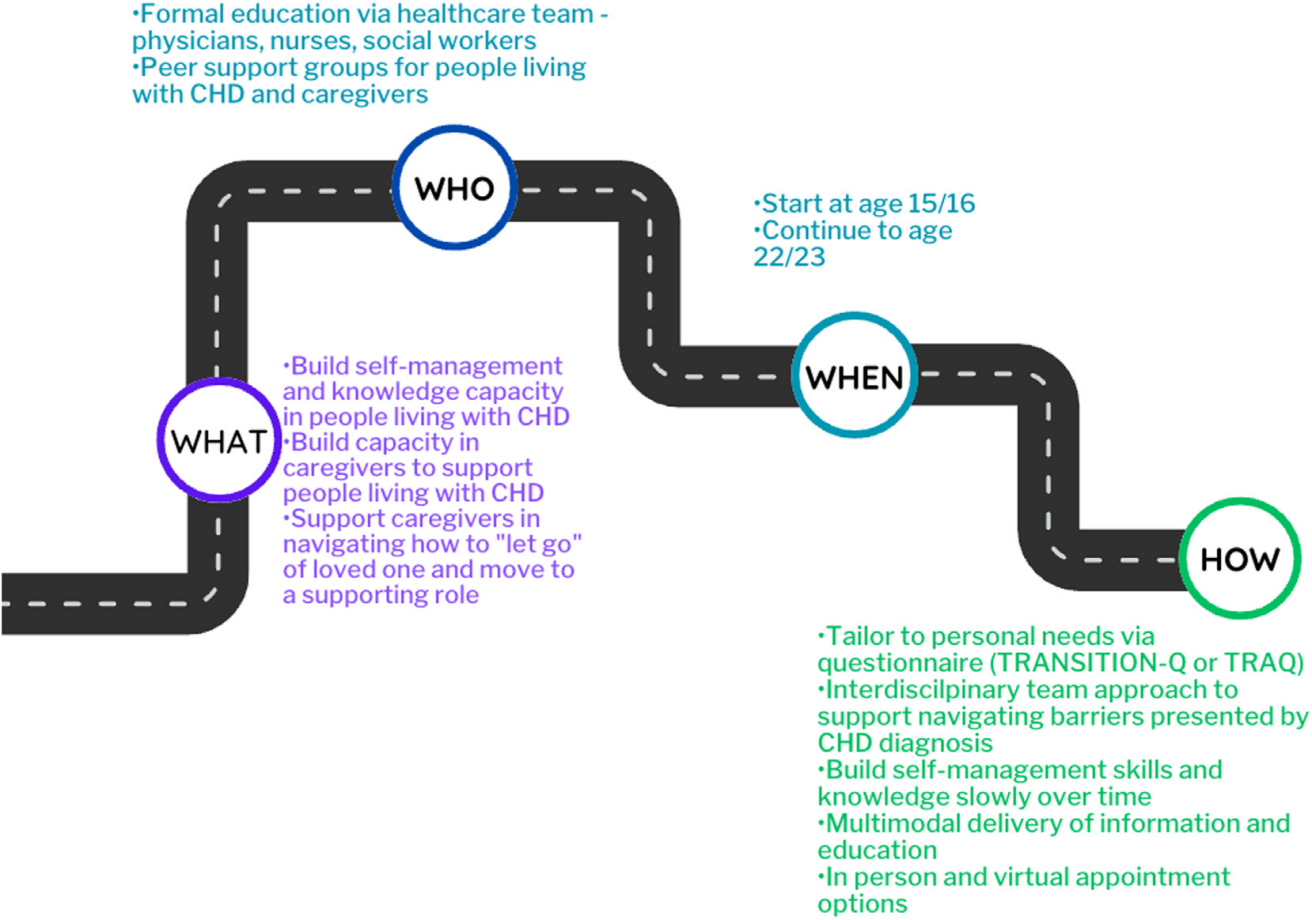
A summary of the results. CHD, congenital heart disease; TRAQ, Transition Readiness Assessment Questionnaire.

What emerged from the series of consultation workshops were recommendations, the Transition Essentials, related to all aspects of the transition process. The Transition Essentials recommendations cover The What—what information, education, or support is needed, The Who—who should provide this information, education, or support, The When—when should this information, education, or support be provided, and The How—how should this information, education, or support be provided (Figure 2).

### The What: What information, education, or support?

There was explicit agreement among patients, their caregivers, and health care providers that patients need support to build the capacity to take charge of their health care and life. This was broken down into both self-management and CHD-related knowledge. For self-management, support was noted to be required related to the importance of monitoring symptoms, life-long care, asking questions of the health care team independent of their caregivers, career planning, and lifestyle considerations. For knowledge related to CHD, patients and their caregivers shared that support is required to learn to describe their heart defect and the procedures they have undergone.

What also emerged from the consultation workshops was the importance of coaching caregivers on how to help build capacity in patients and how to carve out a new role for themselves in their loved one’s life as caregivers of an adult rather than a child. The concept of “letting go” was troubling to caregivers, as they had been on this journey with their loved one for 18 years and had been key in providing information and liaising with the health care team. Caregivers desired to give freedom to their loved ones and wanted to build capacity in them to manage their health; however, they noted needing support to do this. They desire information to help them clarify what role they now play in the life of their loved one.

### The Who: who should provide this information, education, or support?

Who should provide the information, education, and support flowed naturally from discussing what information or education is needed. It was clear from the lived and clinical expertise that the health care team needs to provide medical information and education. Patients and their caregivers respect and listen to this group. Caregivers shared that they were happy to reinforce messages; however, they wanted the key information and education from the health care team to come to the family. When discussing the recommendations, the health care providers shared that nurses and social workers were key professions to include when planning a transition programme. Nurses can provide education on how to self-manage, lifestyle considerations, and CHD. Social workers are poised to provide the support needed for patients to carry out self-management and to caregivers to support their shift in roles. For example, if a specific career that is of interest to a patient is not possible due to their CHD, a social worker can meet with them and support them in identifying what it is about that career that is of interest and help them find career options that meet that interest. Social workers can also support caregivers in their transition of “letting go” and help them learn how to support their loved ones.

Peer support was explored and was an interest among all stakeholder groups. Caregivers believed that peer support would be beneficial in sharing tips on building capacity and being a place to go to discuss concerns with someone who “has walked in my shoes,” to quote one caregiver. Patients agreed that peer support would be beneficial if the focus were not on providing information or education but on providing a place to connect with others who can relate to the specific nuances of living with CHD, such as the lifestyle considerations and limitations that people who do not live with CHD do not understand. This would also be a venue to connect with others living with CHD who are older and who can share lived experiences and provide a safe place to ask questions or provide a peer voice of reason. One adult patient shared that having an older peer to connect with related to alcohol use would have provided a unique perspective and place to learn from the experiences of someone who has “walked in his shoes.” Health care providers believed that peer support was important as a forum to share insights and experiences and build a community among patients and their caregivers.

### The When: when should the information, education, or support be provided?

There was consensus from the lived and clinical expertise that transition-related care should start at the age of 15 or 16 (or when the patient enters high school) and continue to the age of 22 or 23. The age of 15 or 16 was recommended as the starting age as it provides time to build capacity but is close enough that the transition does not seem so far away. Starting earlier may remove the reality of the transition, and recognizing that CHD is only one part of the busy lives of patients and caregivers reduces the priority to focus on transition-specific topics. The suggestion to consider the time of entering high school was specific to discussions related to alcohol and substances. Several patients noted that they were exposed to situations related to alcohol and substance use when entering high school, which they did before age 15 or 16. Continuing to age 22 or 23 was agreed to be important to continue to build capacity when the context of care has changed with the transfer to adult care. Adult patients described a sense of a shift in reality and feeling overwhelmed from the age of 18 to 22 and stated that continued support would be key. This was sensed by health care providers, who believed that ongoing support beyond the age of transfer is required to ensure that the CHD transition is successful. The health care providers also desired the time and ability to provide support beyond the age of transfer as they noted that the transition in CHD care is often only one transition occurring in the lives of patients. The age of transfer is often a time of beginning postsecondary education or entering the workforce, a change in living arrangements, and increased independence. Recognizing this, the health care providers believed that extending the time of transition-specific care would improve both the success and the experience of the transition.

### The How: How should the information, education, or support be provided?

Rather than a formal, structured transition programme, patients and their caregivers emphasized the importance of tailoring a transition programme to the needs of the specific person and family. Although key elements of self-management, knowledge, and support are required by all patients, the specific needs, strengths, areas of opportunity, and capacity differ. All stakeholders agreed that using a questionnaire like the TRANSITION-Q^21^ or the Transition Readiness Assessment Questionnaire^22^ provides insights into what is required to self-manage and identify areas of weakness and strength. These insights allow patients, their caregivers, and their health care providers to work together to develop an individualized care plan. Some caregivers suggested that a questionnaire worded from their perspective (eg, “I encourage my loved one to…”) would support them in ensuring that they are stepping back by providing an opportunity for reflection. The health care providers appreciated this tangible recommendation of how to individualize care. They appreciated that the questionnaire results could be tracked over time to identify growth and areas for continued support.

Patients shared that, while they understood the need for limitations due to their heart defect, this led to mental health struggles, including feelings of frustration, isolation from their peers, and a sense of “now what?.” When discussing limitations with health care providers, an interdisciplinary team approach was devised, including physicians, nurses, and social workers. Each of these interdisciplinary team members has a specific role to play in identifying and navigating limitations. Physicians would identify the limitations and explain the rationale. Nurses would provide support around education and symptom management. Social workers would provide support through conversation, counselling, and connections to community programmes to support patients and their caregivers. An analogy of having a team walk alongside the patient to help them find their way around barriers to safely achieving their goals was agreed upon by all stakeholders.

When discussing how to provide information or education, 1 patient used the concept of building blocks. Giving small amounts of information and education at a time and then building off that to grow capacity and knowledge over time was suggested. Starting with self-management items such as answering a physician’s questions and providing updates on symptoms at the age of 15 or 16 and building up to making appointments at the age of 18 was suggested as a possible progression. Health care providers and caregivers quickly referenced the stages of development during adolescence and emphasized the need to consider these when planning appropriate self-management items. For example, 1 health care provider noted that it was not fair to expect a 15-year-old to schedule their appointments; however, it was fair for them to answer a physician’s questions independently from their caregiver. A similar conversation occurred related to knowledge of CHD. All stakeholders agreed, for example, that it is appropriate to begin with a simple description of the heart defect that the person is living with and build on this over time to ensure that the expectations of the ability to describe the heart defect align with the developmental stage of the person.

All stakeholders suggested multimodal delivery of information and education. Rather than relying solely on a website, providing written material was suggested to allow for quick and easy access and access for those living in areas with poor internet connection. All stakeholders agreed that they liked the messaging style of the I Heart Change website,^23^ specifically that information was shared honestly and clearly. The ability to ensure that information is accurate and up-to-date was emphasized by 1 caregiver, which is an important consideration in ensuring the sustainability of a website. Specific to the MyTransition app, the ability to store a description of their specific heart defect and their health care providers’ names and contact information was appealing. The patients and their caregivers wished to be able to complete a questionnaire on the app and have this information shared with the health care team automatically. The health care providers agreed and recommended saving the questionnaire in the patient’s health record. Other expansions of the app’s functionality included more education information or links to a website housing the information and reminders of upcoming appointments or when appointments are due. One patient, who is attending postsecondary education outside of the province, and their caregiver suggested having a standardized website and app for use by all CHD programmes across the country to ensure consistency in messaging and information being shared.

Finally, all stakeholders endorsed options for both in-person and virtual transition-specific appointments. Patients envisioned these appointments occurring outside their usual CHD-specific care. Still, they noted that sitting down with someone in person can be beneficial in having conversations and sharing important information. Equally beneficial was connecting virtually, allowing appointments to fit more easily into busy schedules. In-person appointments were specifically suggested by patients if adult CHD care was provided at a new hospital site. This visit could include a guided introduction to the new space in which patients with CHD will receive care. Health care providers appreciated the ability to provide virtual care options to reduce travel requirements while increasing the touchpoints between the health care team and people living with CHD.

## Discussion

We designed a series of consultation workshops attended by patients, their caregivers, and CHD health care providers to co-develop recommendations that could be applied to informing the design of a transition programme from paediatric to adult specialized CHD care. The resultant recommendations, which we named the “Transition Essentials,” describe *what*, *who, when,* and *how* information, education, or support should be provided. Incorporating stakeholder-driven recommendations, such as the Transition Essentials, into a transition programme is an important step towards providing individualized care throughout the transition, which may better meet the needs of patients and their caregivers. This patient-centred approach to health care service redesign may improve both the success of transitioning and continuity of care, as the literature suggests that a large proportion of patients are lost to follow-up^5^ and literature also indicates that continued specialty CHD care improves patient survival.^24^

To our knowledge, this is the first study to approach the development of a transition programme from paediatric to adult CHD care through the engagement of patients and their caregivers. See Box 2 for the impacts of engagement. In contrast, 4 studies were developed without any input from those receiving and experiencing the care.^7^, ^8^, ^9^^,^^25^ One study is positioned more similarly to ours, as the authors used the qualitative study findings to explore the needs, attitudes, and experiences of patients and their caregivers to design the transition programme independently.^26^ Although this study included the experiences of patients and their caregivers, it is limited to the data gathered and interpreted by researchers.^26^ This removes the opportunity for patients and their caregivers to provide insights into components of the transition programme that researchers do not specifically ask about and to provide feedback on whether the operationalization of their suggestions accurately reflected what they had suggested. Health care providers also informed this study, but only through survey responses, again limiting their input to what was specifically asked about.^26^

**Box 2 Impacts of patient engagement**

**Positive**

• Before engaging, the health care team had envisioned a formal, structured transition programme delivered by nursing that provided the same information about self-management and education about congenital heart disease (CHD) to all people living with CHD who attended. This vision was quickly and significantly shifted to focus more on each patient’s needs and expanded to include their caregivers and the additional support required beyond information and education. Throughout each consultation workshop, the concept of being genuinely patient centred, of wrapping care around the person and their caregivers, was emphasized.

**Negative**

• A negative impact of engagement is the time required to ensure meaningful engagement, including developing relationships and ensuring adequate information sharing. Rather than planning and initiating a transition programme over a few months, it took 14 months to complete this project, resulting in the generation of recommendations that will be incorporated into the programme’s design through a separate and iterative participatory process. This extended timeframe must be considered when engaging with people with lived experience, as should the budgetary considerations to compensate them for their time and sharing of their experiences.^35^^,^^36^

The current study aligns with the existing literature on what information and education are needed for patients and their caregivers. Learning to self-manage and gaining knowledge related to CHD were highlighted by all stakeholders as key information and education topics to cover, aligning with existing literature.^2^^,^^6^, ^7^, ^8^, ^9^^,^^25^^,^^27^^,^^28^ Self-management includes making and attending medical appointments, engaging with the health care team directly, and filling and taking prescriptions.^2^ Gaining knowledge related to CHD, such as the topics identified through this current study (ie, name of cardiac condition, reproductive health and family planning, career planning and limitations, and physical exercise), is important as this knowledge has been linked to increased communication between patients and their health care team and with reduced delays in obtaining adult CHD care.^2^ This knowledge can also ensure an understanding the impact of lifestyle choices, such as smoking, alcohol, and recreational drug use specific to CHD. The inclusion of caregivers in transition education is also supported by literature.^7^^,^^28^^,^^29^ Caregivers are transitioning from being solely responsible for their loved one’s health to support their loved one in being independent.^2^^,^^7^^,^^28^^,^^29^ They require support to feel informed and prepared for the transition process and to work with their loved one and the health care team to gradually shift responsibility while ensuring their loved one’s safey.^29^

This study diverges from the existing literature on who is involved in the transition programme, and when and how the transition programme is delivered. The current study supports the existing literature that health care providers are considered the experts by patients and their caregivers.^2^^,^^3^^,^^27^^,^^28^ The current study also reinforces the importance of nursing support for transition CHD programmes.^30^ The divergence in the current study relates to the interdisciplinary team of health care providers that should be included in the transition programme. This is the first study to define a role for social work beyond support for scheduling, insurance, or billing,^31^ despite social work being identified as an institutional requirement for a comprehensive adult CHD centre.^30^^,^^32^ Integrating social work into the interdisciplinary transition team provides an additional important set of skills that can be accessed for support by patients and their caregivers. The interdisciplinary team is also integral to health care delivery, as each member performs a specific role. In the above example, the physician can care for their next patient knowing that the required supports have been made available to the patient through the nurse and social worker.

Regarding when the programme is delivered, 1 existing programme in Belgium starts earlier (age 12)^7^ than the age suggested by the current study. In contrast, others begin at age 15 or 16, as suggested by the current study.^8^^,^^9^^,^^26^ All existing programmes provide transition supports only to the time of transfer^7^, ^8^, ^9^^,^^26^ despite literature supporting continuing the transition programme beyond the age of transfer.^2^^,^^3^ Continuation of transition programming facilitates the continued development of self-management skills and knowledge.^2^^,^^3^ These skills may be part of the survival benefit noted with continued specialty CHD care.^24^ Regarding how the programme is delivered, the current study does not define a specific structure for the transition programme but rather a flexible process. Existing programmes are structured to provide 1,^9^ 2,^8^ 4,^26^ or more^7^ transition-specific appointments. The current study does not define a specific number of transition-specific appointments but rather that the volume and timing of the appointments should be tailored to the needs of each patient. This may be done by annual visits to support the delivery of information and education or more frequent visits to provide additional support, such as in-depth social work support.

### Limitations

This study has some limitations. First, through the workshops, we consulted a relatively small number of patients, caregivers, and health care providers. This reduced the diversity of experiences available to inform this study. That said, the health care providers who engaged in the study encompassed many of the professions involved in paediatric and adult CHD care, physicians, nursing, and social work. Further, the patients and caregivers who engaged in the workshop were meant to represent their experiences as a starting point for the development of the transition programme, so we chose smaller group numbers that allowed room for richer conversations,^33^ knowing that this is the first step of a large engagement process. We did not collect sociodemographic information from the people with lived experience or their caregivers. This aligns with the conceptualization of patient partners as research team members who do not require informed consent.^34^ We recognize the limitation of not tracking and reporting the sociodemographics of patients and caregivers and health care providers and suggest that future studies discuss this limitation with all team members to support an improved assessment of the perspectives that are being reflected in findings. We also did not apply equity and diversity considerations when recruiting stakeholders. The lived experience of people in remote areas, low socioeconomic status, and specific communities may differ significantly from those living in urban centres with many resources. This important limitation is acknowledged and will be rectified in the further co-development of the transition programme. Important considerations such as advanced directives, power of attorney, and delegation of consent were not discussed but are important aspects of transition that should be considered in future work. Also not discussed were ways for communication among the team of health care providers throughout the transition journey. This is an important aspect of the operationalization of the recommendations for future studies. This communication will be context specific, as it will depend on the integration of paediatric and adult documentation, health care providers, and the use of paper or electronic documentation. Planning for appropriate and timely communication will be an important consideration in future implementation work. Finally, the context of the Canadian health care system removes the barrier of insurance from accessing CHD care.

## Conclusions

This study demonstrates that engaging patients, their caregivers, and health care providers in developing essential recommendations for transitions from paediatric to adult CHD care leads to tangible recommendations. It also suggests that consideration of the structure of a transition programme is needed in future studies. In Supplemental Appendix S1, we provide potential future studies that continue to engage patients, caregivers, and health care providers as partners in their design and conduct. Shifting away from inflexible transition programmes and towards providing individualized care may improve the success of transitions. Supporting successful transitions from paediatric to adult CHD care is imperative in reducing patients being lost to follow-up or having a lapse in care^4^^,^^5^ and further improving survival in these patients.^24^
