# Supplementary material for: Multistakeholder Recommendations for Supporting Patients and Families Transitioning From Paediatric to Adult Congenital Heart Disease Care
Source: CJC Pediatr Congenit Heart Dis. 2023 Aug 18;2(5):225–36. doi: 10.1016/j.cjcpc.2023.08.001 (PMC10642106; doi:10.1016/j.cjcpc.2023.08.001)
Supplement: Supplemental Appendix S1 [file mmc1.docx]

**Future Work**

The Transition Essentials require operationalization into practice and evaluation through future studies that continue to engage patients, caregivers, and healthcare providers as partners in their design and conduct. A tailored transition program guided by these recommendations may support people living with CHD and their caregivers to successfully transition since these recommendations are informed by their lived experiences. This aligns the information and education provided with patient and caregiver priorities and preferences and increases the relevance to the individual. Potential next steps include re-engaging people living with CHD, their caregivers, and healthcare providers to guide the identification and/or development of the specific tools that will be used to support this future program, including a website and an app. Determining a skeleton structure and sharing the options available to personalize the transition program are key steps for future work and engagement. People living with CHD and their caregivers must also be engaged in selecting outcome measures. Current transition programs are evaluated by changes in the TRAQ^22^ score^8,9,26^, changes in knowledge^8,9,26^, changes in patient empowerment^26^, and achieving the recommended time between pediatric and adult CHD visits^8^. Including patient-reported outcome measures (PROMs) and patient-reported experience measures (PREMs)^36^ in trials to determine the effectiveness of a transition intervention from a patient’s perspective is important and should be explored in future studies. PROMs are validated questionnaires that measure patient perceptions of health status, level of impairment, disability, and health-related quality of life. PREMs explore the experience of receiving care with a focus on the process of care^36^. Including PROMs and PREMs along with current outcome measures of improved knowledge and self-management skills may provide more robust evidence related to the transition program; however, this needs to be explored with people living with CHD and their caregivers. Completing these steps should result in the co-design of an initial transition program, which will then need to be implemented, evaluated, and likely revised following an iterative and participatory process.
